# Supplementary material for: Mitotic regulators and the SHP2-MAPK pathway promote IR endocytosis and feedback regulation of insulin signaling
Source: Nat Commun. 2019 Apr 1;10:1473. doi: 10.1038/s41467-019-09318-3 (PMC6443781; doi:10.1038/s41467-019-09318-3)
Supplement: Supplementary file 1 — Supplementary Information [file 41467_2019_9318_MOESM1_ESM.pdf]

Mitotic Regulators and the SHP2-MAPK Pathway Promote IR Endocytosis and Feedback Regulation of Insulin Signaling

Choi et al.

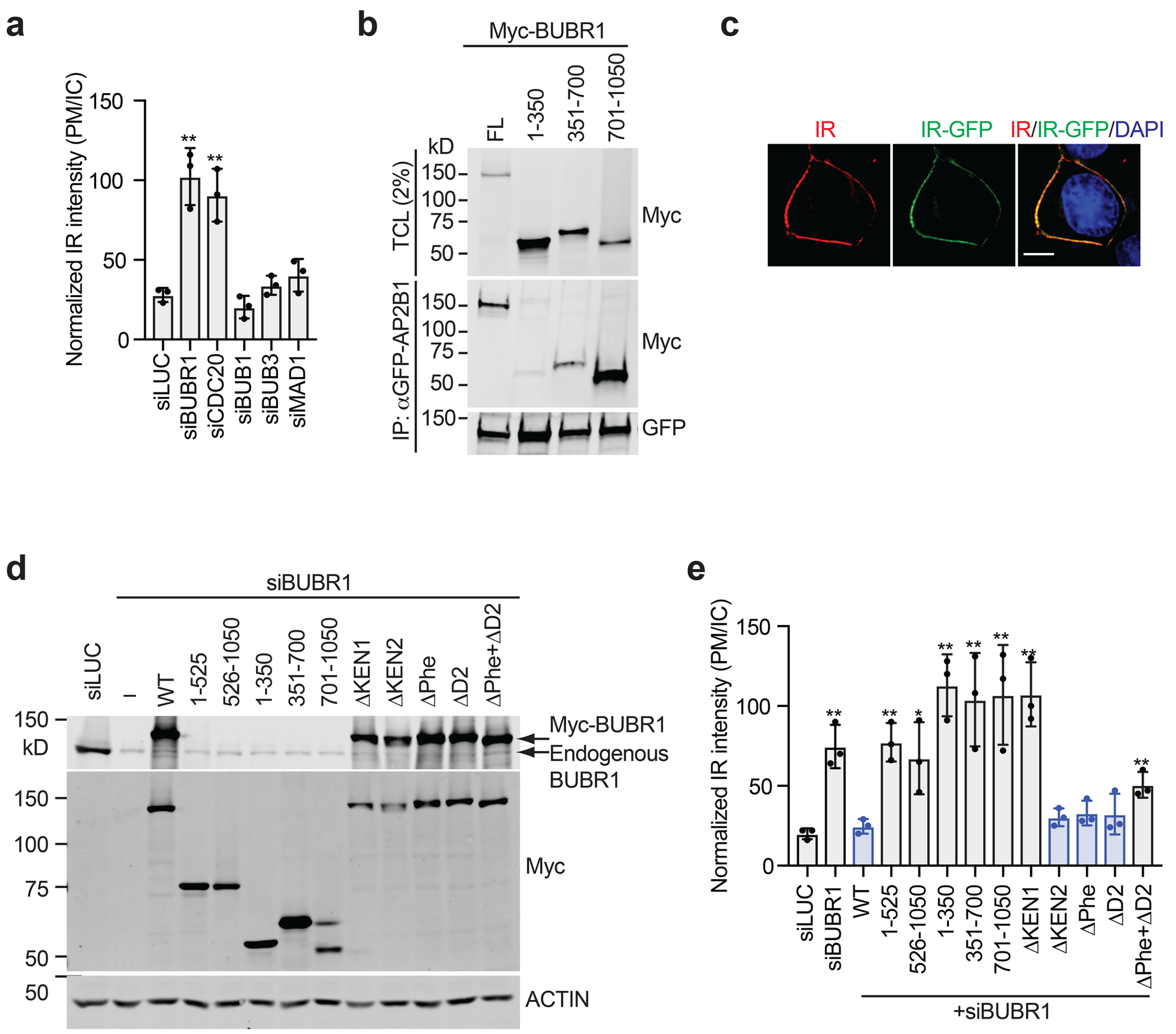

Supplementary Fig. 1 The BUBR1–AP2B1 interaction is critical for IR endocytosis. **a** Quantification of the relative levels of insulin-activated IR endocytosis of cells in Fig. 1f. The ratios of PM and IC IR-GFP signals of insulin-treated cells were divided by those of untreated cells and plotted (mean ± s.d.; **p<0.01; two-tailed unpaired t test; n= 3 independent experiments). **b** Binding of BUBR1 full length (FL) and truncation mutants to AP2B1. 293FT cells were transfected with plasmids encoding GFP-AP2B1 and Myc-BUBR1 proteins. Total cell lysate (TCL) and anti-GFP-AP2B1 IP were blotted with anti-Myc and anti-GFP antibodies. **c** 293FT cells stably expressing IR-GFP WT were serum starved and stained with anti-GFP and anti-IR antibodies. Scale bar, 5 µm. **d** Western blot analysis of lysates of cells in Fig. 2d. **e** Quantification of the relative levels of insulin-activated IR endocytosis of cells in Fig. 2e. The ratios of PM and IC IR-GFP signals of in insulin-treated cells were divided by those in untreated cells and plotted (mean ± s.d.; *p<0.05 and **p<0.01; two-tailed unpaired t test; n= 3 independent experiments). Source data are provided as a Source Data file.

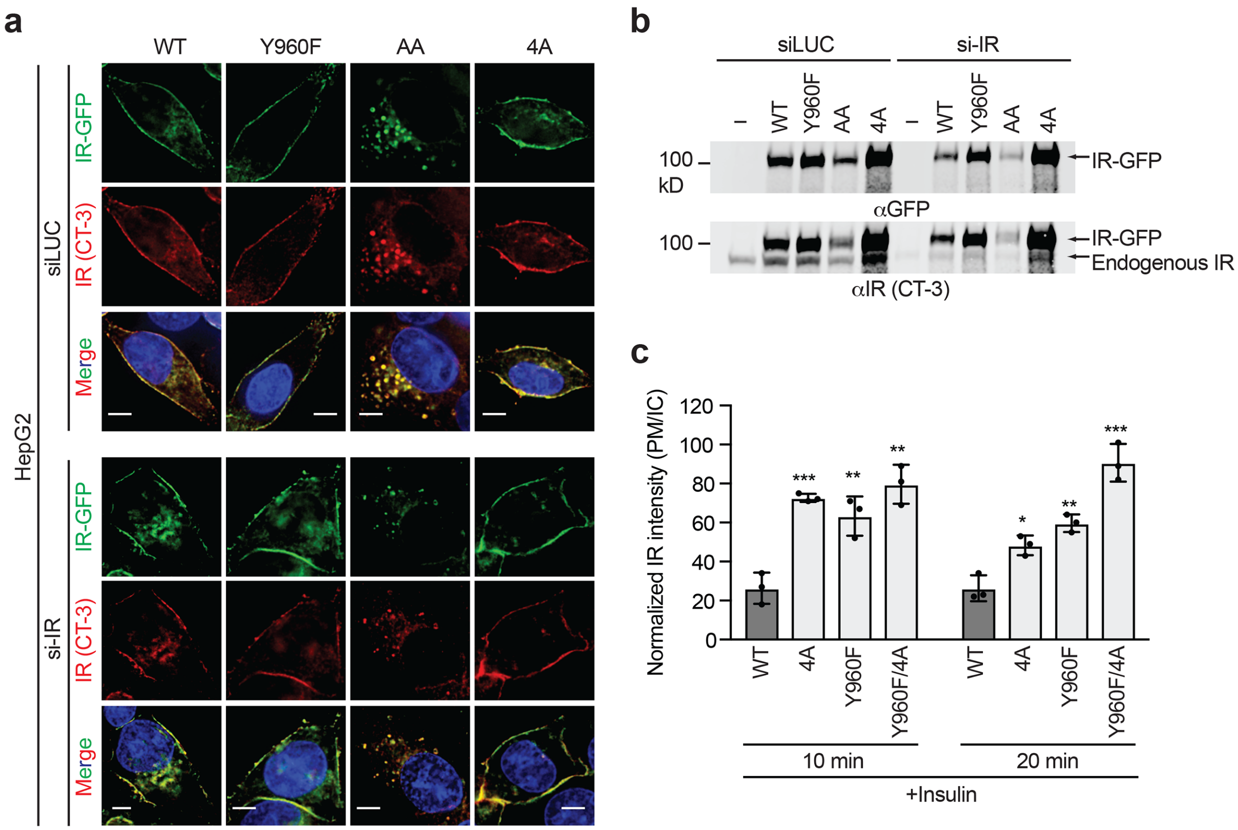

Supplementary Fig. 2 Functions of different IR motifs in the localization and endocytosis of IR. **a** HepG2 cells stably expressing IR-GFP WT, 4A, Y960F, or AA were transfected with the indicated siRNAs, serum starved, and stained with anti-GFP and anti-IR antibodies. Note that the anti-IR antibody detects both the endogenous IR and IR-GFP. Scale bars, 5 µm. **b** Western blot analysis of lysates of cells in (**a**). **c** Quantification of the relative levels of insulin-activated IR endocytosis of cells in Fig. 3f. The ratios of PM and IC IR-GFP signals of insulin-treated cells were divided by those of untreated cells and plotted (mean ± s.d.; *$p<0.05$, **$p<0.01$, and ***$p<0.001$; two-tailed unpaired t test; n= 3 independent experiments). Source data are provided as a Source Data file.

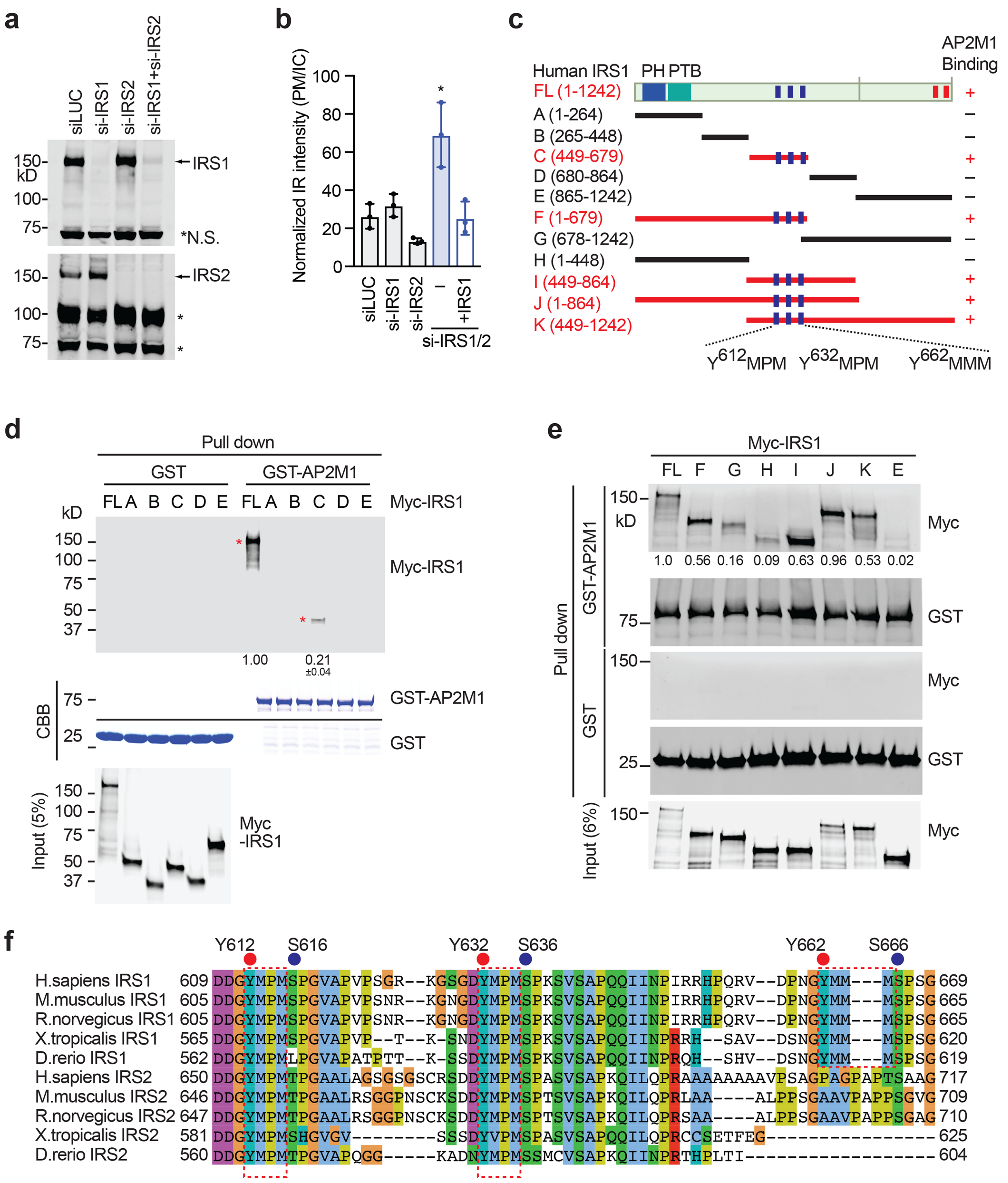

Supplementary Fig. 3 IRS1 promotes IR endocytosis and interacts with AP2. **a** Western blot analysis of cell lysates in Fig. 4a. Asterisks indicate non-specific bands. **b** Quantification of the relative levels of insulin-activated IR endocytosis of cells in Fig. 4b. The ratios of PM and IC IR-GFP signals of insulin-treated cells were divided by those of untreated cells and plotted (mean ± s.d.; *p<0.05; two-tailed unpaired t test; n= 3 independent experiments). **c** Domains and YXXΦ motifs of human IRS1. PH, pleckstrin homology domain; PTB, phosphotyrosine-binding domain.

IRS1 fragments that can or cannot bind to AP2M1 are presented as red or black lines, respectively. YXXΦ motifs and phosphotyrosine sites for SHP2 binding are presented as blue and red bars, respectively. **d** Binding of IRS1 WT and mutants to GST or GST-AP2M1. Input and protein bound to beads were blotted with anti-Myc (IRS1) antibodies and stained with Coomassie (CBB). The relative band intensities are shown below (mean ± s.d.; n=3 independent experiments). **e** Binding of IRS1 WT and truncation mutants to GST or GST-AP2M1. Input and protein bound to beads were blotted with the indicated antibodies. The relative band intensities are shown below (n=2 independent experiments). **f** Sequence alignment of a conserved region in IRS1/2. Three YXXΦ motifs are boxed with red dashed lines. The phosphorylation sites of IR and MAPK are indicated as red and blue dots, respectively. Source data are provided as a Source Data file.

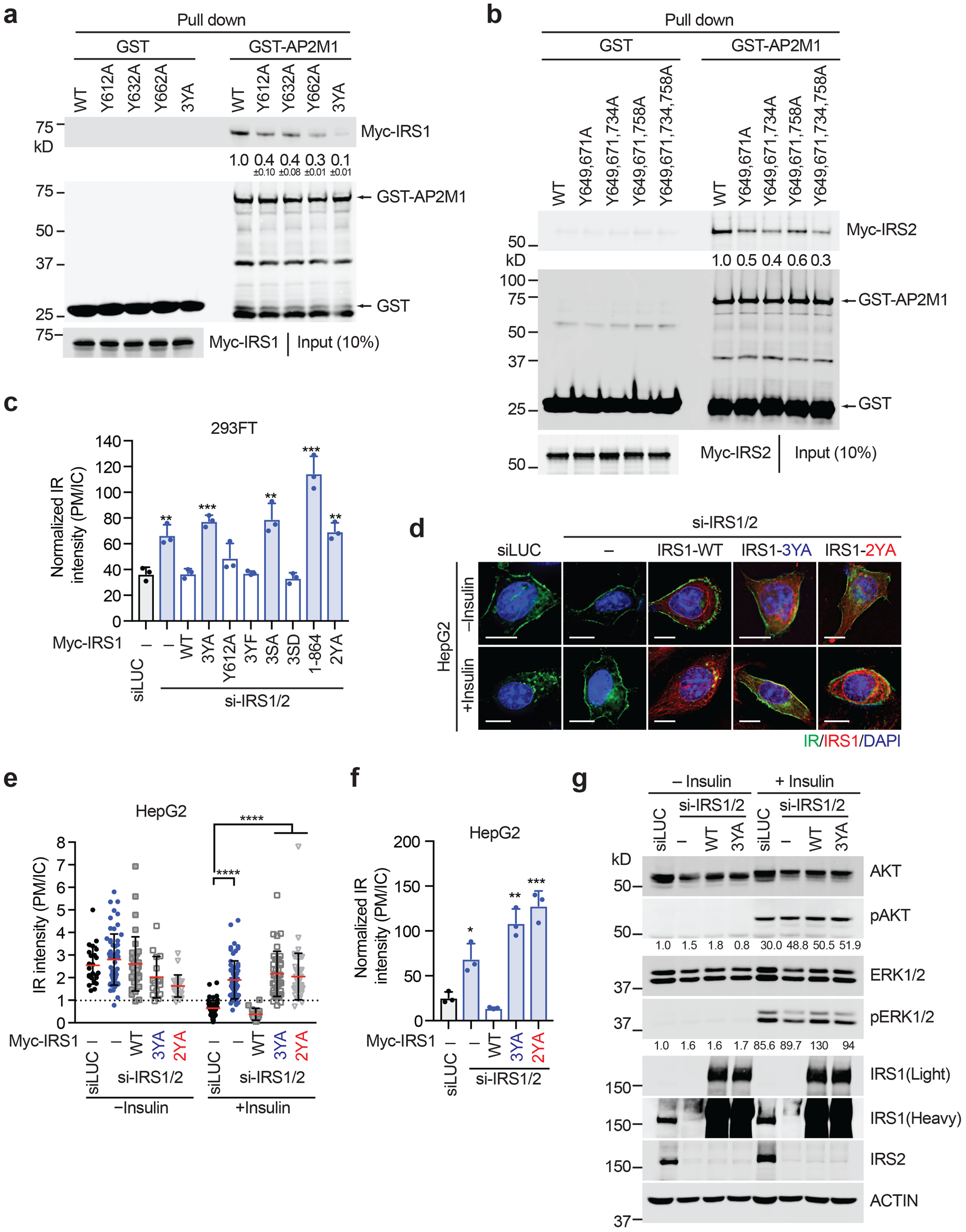

Supplementary Fig. 4 The YXXΦ motifs of IRS1/2 bind to AP2M1 and are required for insulin-activated IR endocytosis. **a** Binding of IRS1 WT and mutants to GST or GST-AP2M1. The relative

band intensities are shown below (3YA, the IRS1 Y612A/Y632A/Y662A triple mutant; mean ± s.d.; n=3 independent experiments). **b** Binding of IRS2 WT and mutants to GST or GST-AP2M1. The relative band intensities are shown below (Mean; n=2 independent experiments). **c** Quantification of the relative levels of insulin-activated IR endocytosis of cells in Fig. 4e. The ratios of PM and IC IR-GFP signals of in insulin-treated cells were divided by those in untreated cells and plotted (mean ± s.d.; $^{**}p<0.01$ and $^{***}p<0.001$; two-tailed unpaired t test; n= 3 independent experiments). **d** HepG2 cells stably expressing IR-GFP WT were transfected with the indicated siRNA or siRNA-resistant mCherry-IRS1, serum starved, treated without or with 100 nM insulin for 5 min, and stained with anti-GFP (IR; green), anti-mCherry (IRS1; red), and DAPI (blue). (3YA, the IRS1 Y612A/Y632A/Y662A triple mutant; 2YA, the Y1179A/Y1229A double mutant). Scale bar, 5 μm. **e** Quantification of the ratios of PM and IC IR-GFP signals of cells in (**d**) (mean ± s.d.; $^{****}p<0.0001$; two-tailed unpaired t test). **f** Quantification of the relative levels of insulin-activated IR endocytosis of cells in (**e**). The ratios of PM and IC IR-GFP signals of insulin-treated cells were divided by those of untreated cells and plotted (mean ± s.d.; $^{*}p<0.05$, $p^{**}<0.01$, and $p^{***}<0.001$; two-tailed unpaired t test; n= 3 independent experiments). **g** 293FT cells stably expressing IR-GFP WT were transfected with the indicated siRNA or siRNA-resistant Myc-IRS1, serum starved, treated without or with 100 nM insulin for 5 min. The cell lysates were subjected to quantitative immunoblotting with the indicated antibodies. The relative band intensities are shown below. Representative results from 3 independent experiments were presented. Source data are provided as a Source Data file.

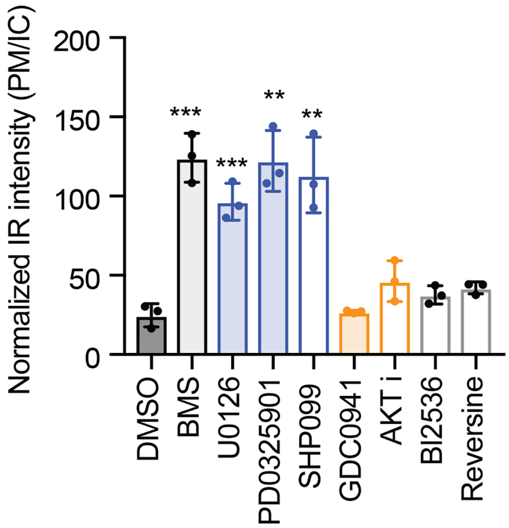

Supplementary Fig. 5 Inhibitors of MEK and SHP2 attenuate insulin-activated IR endocytosis. Quantification of the relative levels of insulin-activated IR endocytosis of cells in Fig. 5b. The ratios of PM and IC IR-GFP signals of in insulin-treated cells were divided by those in untreated cells and plotted (mean ± s.d.; $p^{**}<0.01$ and $p^{***}<0.001$; two-tailed unpaired t test; n= 3 independent experiments). Source data are provided as a Source Data file.

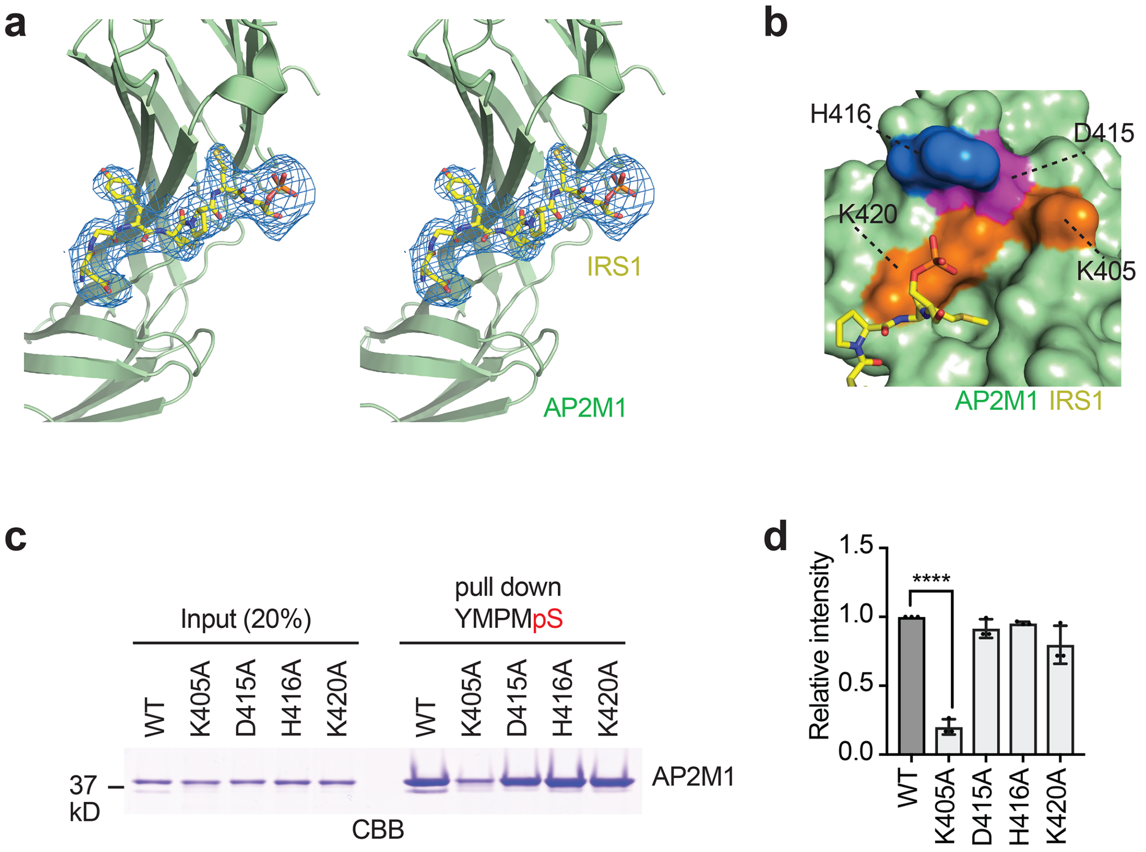

Supplementary Fig. 6 Binding of the pS-IRS1 peptide to AP2M1. **a** Stereo view of the electron density of the IRS1 peptide, which is shown as sticks. The 2*F*o-*F*c map is contoured to 1.0 σ and shown as a blue mesh. **b** Surface drawing of AP2M1 with the bound pS-IRS1 shown as sticks. The potential acceptor residues for IRS1 pS616 are labeled. **c** Binding of the pS-IRS1 peptide to WT and mutants of AP2M1 (residues 160-435). Input and proteins bound to pS-IRS1 peptides were analyzed by SDS-PAGE and stained with Coomassie (CBB). **d** Quantification of the relative band intensities in (**c**) (Mean ± s.d.; $p^{****}<0.0001$; two-tailed unpaired t test; n=3 independent experiments). Source data are provided as a Source Data file.

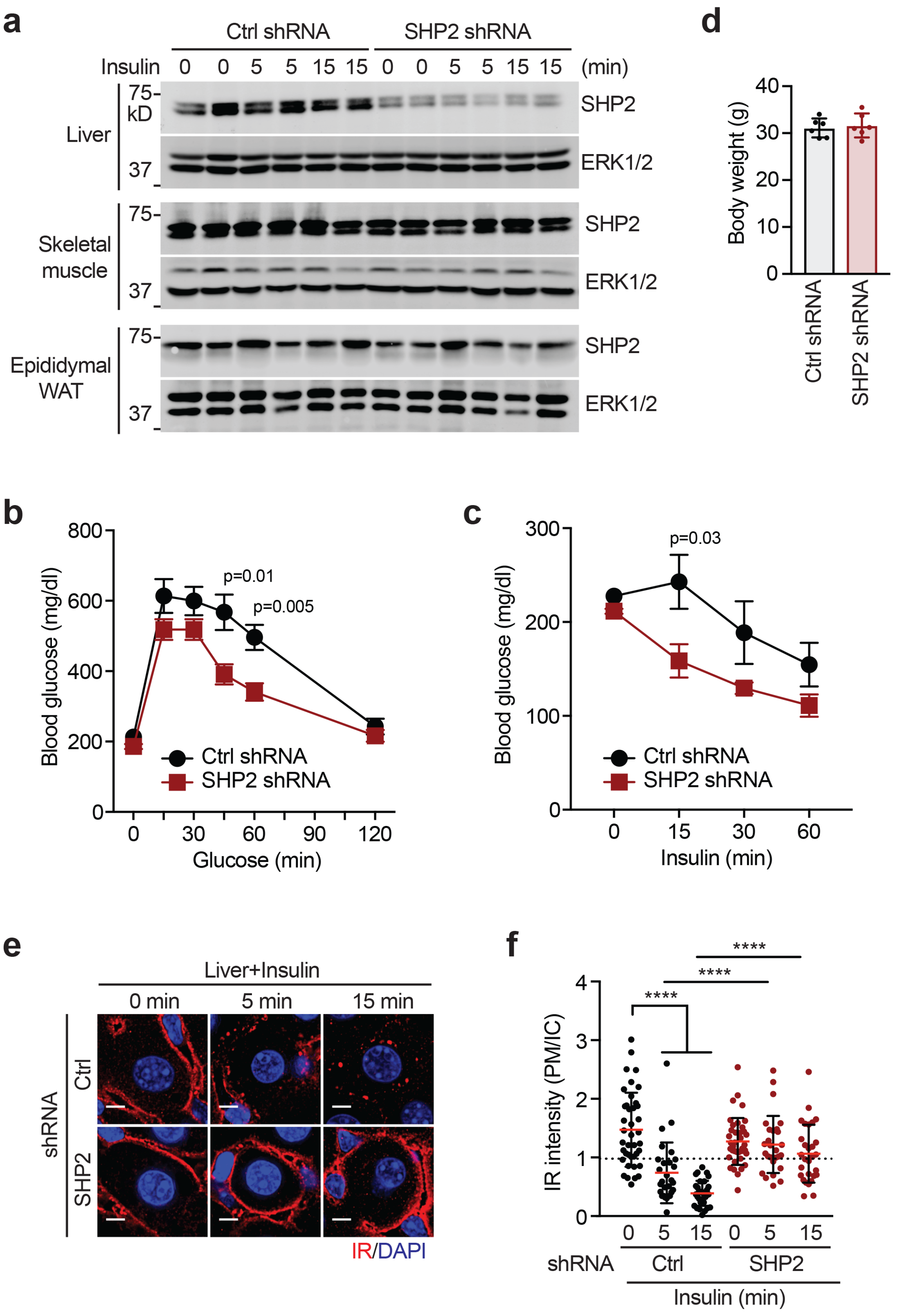

Supplementary Fig. 7 Depletion of SHP2 by shRNA delays IR endocytosis and improves insulin sensitivity in mice. **a** The level of SHP2 in liver, skeletal muscle and epididymal WAT from mice

fed HFD for 5 weeks. The mice were injected with AAV-control (Ctrl) or SHP2 shRNA. At 17 days after injection, the mice were fasted overnight and injected with or without 1 U insulin via inferior vena cava. The livers were collected at the indicated time points. WAT and skeletal muscle were collected at 2 min and 3 min after the indicated time points, respectively. Lysates were prepared from these tissues and subjected to quantitative immunoblotting with the indicated antibodies. **b**,**c** Glucose tolerance test (**b**) and insulin tolerance test (**c**) in mice injected with AAV-Ctrl or AAV-SHP2 shRNA and fed HFD. Experiments were performed at 2 weeks after injection. n=6; mean ± s.e.m. **d** Body weight in HFD-fed mice injected with AAV-Ctrl or AAV-SHP2 shRNA. Mean ± s.d. Ctrl shRNA, n=6; SHP2 shRNA, n=6. **e** HFD-fed mice were injected with AAV-Ctrl or AAV-SHP2. At 17 days after injection, the mice were fasted overnight and injected with or without 1U insulin via inferior vena cava. The livers were collected at the indicated time points and the sections were stained with anti-IR (red) and DAPI (blue). Scale bars, 5 µm. **f** Quantification of the ratios of PM and IC IR signals of the livers in (**e**) (ctrl shRNA, 0 min, n=40; 5 min, n=28; 15 min, n=32 and SHP2 shRNA, 0 min, n=39; 5 min, n=27; 15 min, n=29; mean ± s.d.; ****$p<0.0001$; two-tailed unpaired t test). Source data are provided as a Source Data file.

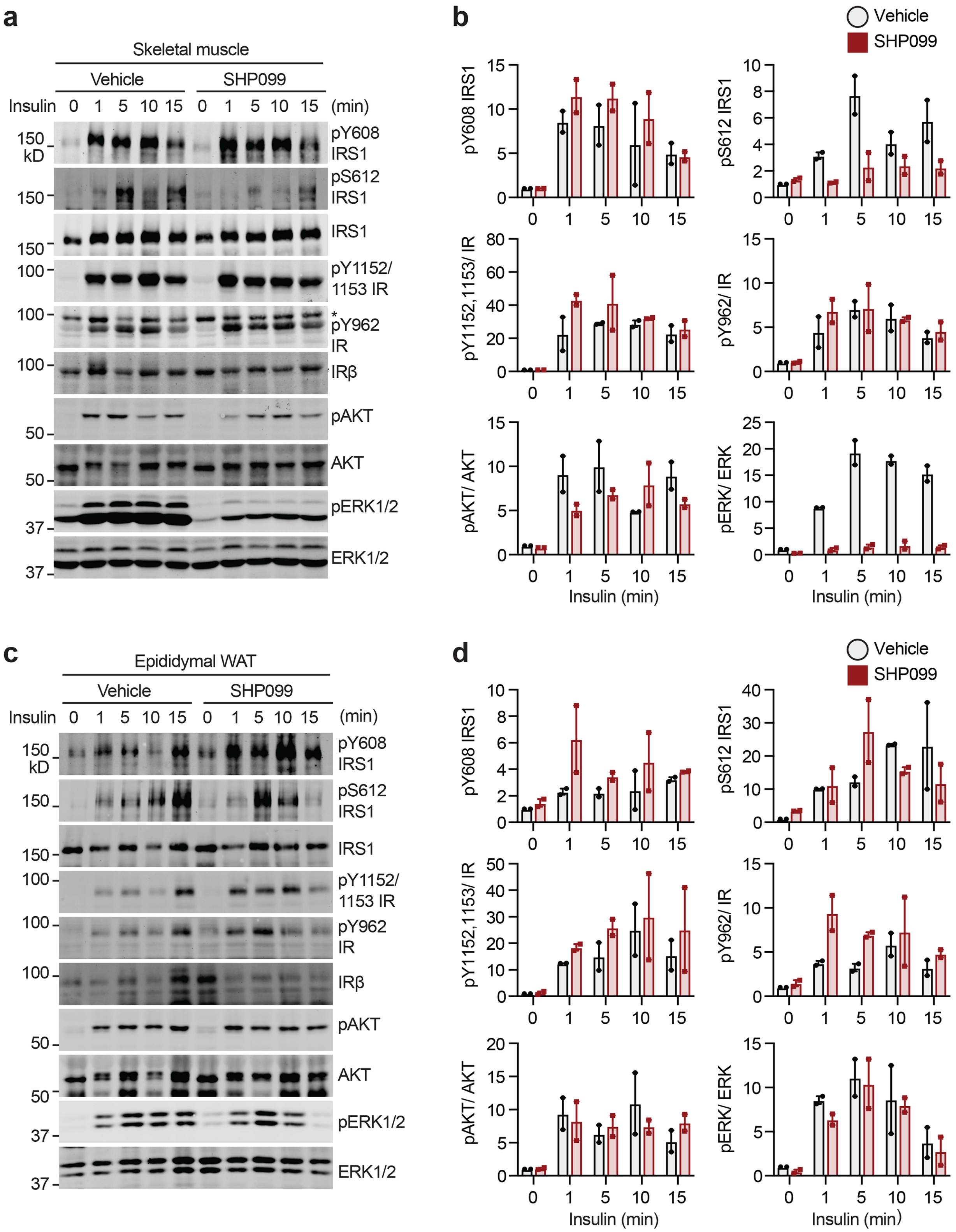

Supplementary Fig. 8 Effect of SHP099 on insulin signaling in skeletal muscle and white adipose tissue (WAT) from HFD-fed mice. **a** Insulin signaling in the skeletal muscle from mice fed HFD

for 5 weeks. The mice were administered vehicle or SHP099 for 5 days, fasted overnight and administered vehicle or SHP099 once more. At 2 h after the last administration, the mice were injected with or without 1 U insulin via inferior vena cava. The livers were collected at the indicated time points (See Fig. 8a,b). WAT and skeletal muscle were collected at 2 min and 3 min after the indicated time points, respectively. Lysates were prepared from these tissues and subjected to quantitative immunoblotting with the indicated antibodies. Asterisk indicates a non-specific band. **b** Quantification of the blots in (**a**). Mean ± range; n=2 independent experiments. **c** Insulin signaling in the WAT from mice in (**a**). **d** Quantification of the blots in (**c**). Mean ± range; n=2 independent experiments. Source data are provided as a Source Data file.

Supplementary Table 1 List of primers used in this study

Name	Sequence
AP2B1_Fse1_For	agc GGCCGGCC T ATG ACT GAC TCC AAG TAT TTC AC
AP2B1_Asc1_Rev	GAG GGCGCGCC TTA GTT TTT CAA AAT GCT GTC GT
AP2M1_Fse1_For	agc GGCCGGCC T ATG ATT GGA GGC TTA TTC ATC TA
AP2M1_Asc1_Rev	GAG GGCGCGCC CTA GCA GCG AGT TTC ATA AAT GC
AP2B1_2486bp_For	CTTCAGCTGCCTCATCCCACTCAA
AP2M1_900bp_For	CtGGAGGtCAAGGtGGtCAtCAA
AP2B1_A(1-280)_Rev_Asc1	GAtGGCGCGCCttACAGCAtAttGtAGtAGtCAGAA
AP2B1_B(280-590)_For_Fse1	TCGGGCCGGCCTCtGAAGAAGttAGCCCCtCC
AP2B1_B(280-590)_Rev_Asc1	GATGGCGCGCCTTAACGATGAATTCCATGACTTC
AP2B1_C(590-700)_For_Fse1	TCGGGCCGGCCTAAACACttGCCAAttCAtCAt
AP2B1_C(590-700)_Rev_Asc1	GAtGGCGCGCCttAGACCACAGCAGGtGtAGGtGAA
AP2B1_D(700-951)_For_Fse1	TCGGGCCGGCCTAGCAGtGGACtGAAtGACCtGt
EC_IR_Y999F_only_F	ttCAAACCCtGAGtttCtCAGtGCCAGtGAtGtG
EC_IR_Y999F_only_R	CACAtCACtGGCACtGAGAAACtCAGGGtttGAA
IR_LL_AL single_For	tCGAGAGAAGAtCACCGCCCttCGAGAGCtGGGg
IR_LL_AL single_Rev	CCCCAGCtCtCGAAGGGCGGtGAtCttCtCtCGA
IR_LL_AA_For	AGAGAAGAtCACCGCCGCtCGAGAGCtGGGGCAGGG
IR_LL_AA_Rev	CCCtGCCCCAGCtCtCGAGCGGCGGtGAtCttCtCt
EC_hIRS1_1_Fse1_F	TCA GGCCGGCC T AtGGCGAGCCCtCCGGAGAGCG
EC_hIRS1-264_Asc1_R	AGA GGCGCGCC ttAGAACtCAtCACtCAtGGCCC
EC_hIRS1_264_Fse1_F	TCA GGCCGGCC T CGCCCtCGCAGCAAGAGCCA
EC_hIRS1_448_Asc1_R	AGA GGCGCGCC ttAAtCCGGAGtGACACtGCGGAA
EC_hIRS1_449_Fse1_F	TCA GGCCGGCC T tCCCtGGGCCACACCCCACCAG
EC_hIRS1_679_Asc1_R	AGA GGCGCGCC ttAGGGGCCACCtCCAAtGtCAGG
EC_hIRS1_680_Fse1_F	TCA GGCCGGCC T AGCAGCAGCAGCAGCAGCAGC
EC_hIRS1_864_Asc1_R	AGA GGCGCGCC ttACCCCAGGGACAGCCtCGtGGG
EC_hIRS1_865_Fse1_F	TCA GGCCGGCC T GAtCCCAAGGCCAGCACCttAC
EC_hIRS1_1242_Asc1_R	AGA GGCGCGCC CtACtGACGGtCCtCtGGCtGCtt
EC_hIRS1_Asc1_deletion_F	AGGCtGGGGGCCCGGCACGCCUCGAGtACtAC
EC_hIRS1_Asc1_deletion_R	GTAGTACTCGAGGCGTGCCGGGCCCCCAGCCT
EC_IRS1_678_F	TCA GGCCGGCC T GGCCCCAGCAGCAGCAGCAGCAGCAGCAAC
EC_IRS1_1242_R_new	AGA GGCGCGCC CtACtGACGGtCCtCtGGCtGCttCtGG
EC_IRS1_4_AMPM_F	GAAAGGGCAGtGGAGACGCtAtGCCCAtGAGCCC
EC_IRS1_4_AMPM_R	GGGCtCAtGGGCAtAGCGtCtCCACtGCCCtttC
EC_IRS1_3_AMPM_F	CACACGGAtGAtGGCGCCAtGCCCAtGtCCCCAGG
EC_IRS1_3_AMPM_R	CCtGGGGACAtGGGCAtGGCGCCAtCAtCCGtGtG
EC_IRS1_5_AMPM_F	GAGtGGACCCCAAtGGCGCCAtGAtGAtGtCCCC
EC_IRS1_5_AMPM_R	GGGGACAtCAtCAtGGCGCCAttGGGGtCCACtC
EC_IRS1_YA_Seq_F	AGtCCtCAGtGGCttCCAttGAGGAGtA
EC_IRS1_678_New_F	TCA GGCCGGCC T GGCCCCAGCAGCAGCAGCAGCAGCAACGCCGt
EC_IRS1_S616A_F	AtGCCCAtGGCCCCAGGGGtGGCCCCAGtGCCCAG
EC_IRS1_S616A_R	CtGGGCACtGGGGCCACCCCtGGGGCCAtGGGCAt
EC_IRS1_S636A_F	AtGCCCAtGGCCCCCAAGAGCGtAtCtGCCCCACA
EC_IRS1_S636A-R	TGtGGGGCAGAtACGCtCttGGGGGCCAtGGGCAt
EC_IRS1_S666A_F	AtGAtGAtGGCCCCCAGCGGtGGCtGCtCtCCtGA
EC_IRS1_S666A_R	tCAGGAGAGCAGCCACCGCtGGGGGCCAtCAtCAt
EC_IRS1_S616D_F	AtGCCCAtGGACCCAGGGGtGGCCCCAGtGCCCAG
EC_IRS1_S616D_R	CtGGGCACtGGGGCCACCCCtGGGtCCAtGGGCAt
EC_IRS1_S636D_F	AtGCCCAtGGACCCCAAGAGCGtAtCtGCCCCACA

EC_IRS1_S636D-R	tGtGGGGCAGAtACGCtCttGGGGtCCAtGGGCAt
EC_IRS1_S666D_F	AtGAtGAtGGACCCCAGCGGtGGCtGCtCtCCtGA
EC_IRS1_S666D_R	tCAGGAGAGCAGCCACCGCtGGGGtCCAtCAtCAt
EC_IRS1_Y616F_F	CCACCCtCCACACGGAtGAtGGCttCAtGCCCAtG
EC_IRS1_Y616F_R	CAtGGGCAtGAAGCCAtCAtCCGtGtGGAGGGtGG
EC_IRS1_Y632F_F	GtGGCCGAAAGGGCAGtGGAGACtttAtGCCCAtG
EC_IRS1_Y632F_R	CAtGGGCAtAAAGtCtCCACtGCCCtttCGGCCAC
EC_IRS1_Y662F_F	CCCAGAGAGtGGACCCCAAtGGCttCAtGAtGAtG
EC_IRS1_Y662F_R	CAtCAtCAtGAAGCCAttGGGGtCCACtCtCtGGG
EC_IRS1_S666D_N_F	CAAtGGCtACAtGAtGAtGGACCCCAGCGGtGGCtGCt
EC_IRS1_S666D_N_R	AGCAGCCACCGCTGGGGTCCATCATCATGTAGCCATTG
EC_New_IRS1_Y662F_F	GtGGACCCCAAtGGCttCAtGAtGAtGtCCCCCAG
EC_New_IRS1_Y662F_R	CtGGGGGACAtCAtCAtGAAGCCAttGGGGtCCAC
EC_AP2M1_K405A_F	tCAAGGtGCGCtACttGGCGGtGtttGAACCGAAGC
EC_AP2M1_K405A_R	GCttCGGttCAAACACCGCCAAGtAGCGCACCttGA
EC_AP2M1_D415A_F	AAGCtGAACtACAGCGCCCAtGAtGtCAtCAAAt
EC_AP2M1_D415A_R	AtttGAtGACAtCAtGGGCGCtGtAGttCAGCtt
EC_AP2M1_H416A_F	GCtGAACtACAGCGACGCtGAtGtCAtCAAAtGGGt
EC_AP2M1_H416A_R	ACCCAtttGAtGACAtCAGCGtCGCtGtAGttCAGC
EC_AP2M1_K420A_F	CGACCAtGAtGtCAtCGCAtGGGtGCGCtACAttGG
EC_AP2M1_K420A_R	CCAATGTAGCGCACCCATGCGATGACATCATGGTCG
EC_AP2M1_1092bp	GCAGAtCAGCGCAGAGAttGAG
EC_mIRS2_520_F_Fse1	TTCAGGCCGGCCTTCAATAGCGGAGACCCCGCCAGCCA
EC_mIRS2_888_R_Asc1	AGAGGCGCGCCTCACTCTAGCGATAGGCGTGTAGGCCG
EC_mIRS2_3AMPM_F	AGATGATGGCgcCATGCCCATGACCCC
EC_mIRS2_3AMPM_R	GCCCCCAGGTTACTGCTG
EC_mIRS2_4AMPM_F	GAGCGATGACgcCATGCCCATGAG
EC_mIRS2_4AMPM_R	TTGCAGCTATTGGGACCA
EC_mIRS2 5 YMPM_F	AGACAGTGGGgccATGCGAATGTGGTGTG
EC_mIRS2 5 YMPM_R	TCTGGGGAGCTCTCCGCT
EC_mIRS2 6 YMPM_F	CAACGGGGACgccCTCAACATGTC
EC_mIRS2 6 YMPM_R	GGGAGTAGCTTAGGGTCT
EC_hIRS1_Y1179A_F	TGGTCTTAACgccATAGACCTGGATTTGGTC
EC_hIRS1_Y1179A_R	TTCTCCAAACCCCCAGCA
EC_hIRS1_Y1229A_F	TTTAAGCGCCgctGCCAGCATCAGTTTCCAG
EC_hIRS1_Y1229A_R	TCCTCACTTGAGCGGCGG
EC_hIRS1_c_seq	TCCTCAACACCCAGTGCCAC
EC_mCherry_F_BamH1	GGCCGGATCCATGGTGAGCAAGGGCGAGGAGGACAAC
EC_mCherry_R_EcoR1	CACTGAATTCCGTCGACGCGGCCGCTTATGGCGCGCCG
